# The Time Course of Person Perception From Voices: A Behavioral Study

**DOI:** 10.1177/09567976231161565

**Published:** 2023-05-25

**Authors:** Nadine Lavan

**Affiliations:** Department of Biological and Experimental Psychology, School of Biological and Behavioural Sciences, Queen Mary University of London

**Keywords:** person perception, voices, time course, gender, traits

## Abstract

Listeners spontaneously form impressions of a person from their voice: Is someone old or young? Trustworthy or untrustworthy? Some studies suggest that these impressions emerge rapidly (e.g., < 400 ms for traits), but it is unclear just how rapidly different impressions can emerge and whether the time courses differ across characteristics. I presented 618 adult listeners with voice recordings ranging from 25 ms to 800 ms in duration and asked them to rate physical (age, sex, health), trait (trustworthiness, dominance, attractiveness), and social (educatedness, poshness, professionalism) characteristics. I then used interrater agreement as an index for impression formation. Impressions of physical characteristics and dominance emerged fastest, showing high agreement after only 25 ms of exposure. In contrast, agreement for trait and social characteristics was initially low to moderate and gradually increased. Such a staggered time course suggests that there could be a temporo-perceptual hierarchy for person perception in which faster impressions could influence later ones.

Upon hearing a voice, listeners spontaneously form a detailed and multivariate impression of the person whose voice they just heard (Lavan, 2023; Lavan & McGettigan, 2023). Consequently, listeners perceive a wide range of person characteristics from the voice, such as a person’s age, gender, body size, traits (e.g., trustworthiness, dominance), and social characteristics (e.g., social class, level of education, occupation; Bayard et al., 2001; McAleer et al., 2014; Moyse, 2014; Owren et al., 2007; Rendall et al., 2007). Surprisingly, however, there is relatively little work on how listeners come to form this wealth of impressions of other people from their voices.

One particular aspect of impression formation that has received little attention to date is the early time course of person perception from voices: How quickly can impressions of different person characteristics be formed? Are all impressions initially established along a similar time course, or are there any differences between the different characteristics? A model of person perception from voices (Lavan & McGettigan, 2023) has recently proposed that, on the one hand, person perception from voices could be a hierarchical or, perhaps, temporally staggered process in which different person characteristics are perceived at different stages of a processing hierarchy. On the other hand, person perception could be a holistic process, in which listeners perceive voices on the basis of meaningful, socially complex percepts (e.g., “sounds like a Valley Girl/city banker”; cf. persona-based accounts in sociolinguistics; see D’Onofrio, 2020, for a review). These two hypotheses could be empirically contrasted by examining the time course of how initial person impressions emerge. A staggered time course, where some impressions emerge more rapidly than others, could indicate that a perceptual hierarchy during person perception exists. Here, earlier impressions could in principle interact with or constrain later impressions. Conversely, largely similar time courses across all characteristics could indicate that listeners initially form a single holistic impression that they can then break down into individual different characteristics, perhaps via top-down processes.

Some relevant insights with regard to these broad predictions can already be gleaned from the existing voice perception literature: Across previous studies, vocal stimuli from a wide range of durations have been sampled, from stimuli presented for less than 1 s (vowels, short words) to recordings of spoken passages of text lasting 30 s or more (cf. McAleer et al., 2014, and Bayard et al., 2001; see also Groyecka-Bernard et al., 2022). Some of these studies have shown that some impressions are already formed after surprisingly little exposure: For example, rating studies show that trait impressions and socially relevant accent impressions can be established from hearing the word “hello” (~400 ms of exposure; Mahrholz et al., 2018; McAleer et al., 2014; Purnell et al., 1999) or after exposure to a single sustained vowel (Rezlescu et al., 2015). There are, however, only two behavioral studies that have formally examined the time course of first-impression formation from voices, including looking for the minimal exposure necessary to form impressions: For trait perception from voices, Mileva and Lavan (2023) established that impressions of traits from vowels are indeed generally formed within 400 ms via a rating task. However, impressions of dominance for male voices emerged more quickly (< 50 ms), and impressions of attractiveness for female voices were also established by 200 ms of exposure. Owren et al. (2007) furthermore examined how accuracy in gender perception from voices evolves over very short exposures. The authors found that above-chance performance for binary gender judgments is already apparent after less than 15 ms of exposure to vowel stimuli. Aside from behavioral studies, electrophysiological studies have shown that gender (Latinus & Taylor, 2012) and selected expressed traits, such as confidence (Jiang & Pell, 2015, 2016a, 2016b) and politeness (Caballero et al., 2021; Vergis et al., 2020), can be differentiated in event-related potentials within a few hundred milliseconds after voice onset. These behavioral and electrophysiological studies of gender and trait perception show that impressions can emerge rapidly after only subsecond exposure to a voice. More systematic research is, however, needed to help us understand both on a theoretical and an empirical level how listeners form their first impressions of other people on the basis of their voices. Specifically, research looking at more than one (or one type of) characteristic within the same study is needed to enable direct comparison of impression formations of different kinds of person characteristics.

**Statement of Relevance**

When we hear another person’s voice, we immediately make up our minds about whom we think this person is in terms of age, gender, or size and what they are like in terms of whether they are friendly, posh, or educated. It is so far, however, unclear how we form such impressions of other people: Do we form all of these different impressions simultaneously, or are some impressions formed more quickly than others? In the current study, I found that adult listeners formed impressions of physical characteristics (e.g., their age, gender, health) before impressions of a person’s social (poshness, level of education) and trait (trustworthiness, attractiveness) characteristics. This means that listeners may first work out, for example, a person’s gender, which could then shape how they will assess the person’s trustworthiness. The current findings thus help us understand how we form first impressions of other people.

In the current study, I therefore set out to examine how quickly impressions of nine person characteristics could be formed from the speaker’s voice. These nine person characteristics were clustered into three broader categories: physical characteristics (age, gender, health), trait characteristics (attractiveness, dominance, trustworthiness), and social characteristics (level of education, poshness, professionalism). To track how and when impressions of person characteristics are initially formed, I ran a gating experiment, during which different groups of listeners rated recordings of 100 talkers producing the vowel /a/ after being exposed to these recordings for either 25 ms, 50 ms, 100 ms, 200 ms, 400 ms, or 800 ms, respectively. On the basis of these ratings, I computed the main measure of interest, the interrater agreement (as measured by the intraclass correlation coefficient [ICC]) of raw ratings for each characteristic and exposure duration. High interrater agreement was seen as evidence that listeners had formed impressions, as in the absence of random responses, listeners must have tapped into a shared representation of what, for example, a trustworthy voice sounds like (see also Mileva & Lavan, 2023). The current study, therefore, measured the initial emergence of shared, entirely subjective impressions based on the sound of a person’s voice. Crucially, the impressions’ relationship to accuracy (i.e., whether impressions are reflective of a person’s true characteristics) or not assessed as it is not of primary interest.

On the basis of the previous literature, I predicted that listeners would have formed initial impressions of all of the characteristics investigated within 400 ms of exposure, following previous reports on the formation of gender, trait, and socially relevant impressions (McAleer et al., 2014; Mileva & Lavan, 2023; Owren et al., 2007; Purnell et al., 1999). Similarly, with studies reporting that gender is perceived more quickly than trait characteristics, a staggered time course can also be expected, where impressions for some characteristics (including gender) may be perceived more quickly than others.

## Open Practices Statement

The raw audio stimuli for this study are publicly accessible at http://www.stimmdatenbank.coli.uni-saarland.de/, and the specific stimuli used are available from the author on reasonable request. The design and analysis plans for this study were not preregistered. Anonymized data (after exclusions) have been made publicly available via OSF (https://osf.io/gfrmh). The analysis code for this study is available from the author on reasonable request.

## Method

### Participants

Data from 618 adult participants were included in the analysis (age: *M* = 40.1 years, *SD* = 12.4; 491 female, one provided no gender data). The sample size for the current study was determined on the basis of previous studies (e.g., McAleer et al., 2014; Mileva & Lavan, 2023) suggesting that approximately 20 to 30 participants per condition are sufficient to measure interrater agreement. In the current study, between 22 and 36 participants were included per condition. For exact sample sizes for each characteristic and exposure duration, see Table S1 in the Supplemental Material available online.

All participants were recruited from Prolific.co, were aged between 18 and 65 years, native speakers of English, and born and currently residing in the United Kingdom. None had any self-reported hearing difficulties. Participants had furthermore completed at least five studies on Prolific.co and had an approval rate of more than 95%, suggesting overall good engagement in the previous studies. The study was approved by the local ethics committee, and participants were paid at least £6 for their participation in the study.

Before arriving at this final participant sample, I excluded a number of participants: One participant was excluded for failing more than 10% of vigilance trials across the entire experiment (27 vigilance trials in total). Thirty-five participants were additionally excluded for using a single response option (e.g., selecting “5” for all experimental trials) for more than 80% of trials on one or more of the rating scales they completed. Finally, one participant was excluded from the analysis of gender for male voices because they used only a single response option, and therefore some measures of interest could not be computed.

### Materials

Recordings of 100 voices (50 male, 50 female) of young adults between 20 and 25 years old were retrieved from the Saarbrücken Voice Database (Pützer & Barry, n.d.). In the voice recordings, talkers produced the vowel /a/ in a sustained manner (> 800 ms in duration) at a comfortable pitch. None of the people in the selected recordings had any diagnosed voice pathologies.

The 100 selected voice recordings were initially normed for their root-mean-square intensity using *Praat* (Boersma & Weenink, 2020) to broadly equalize the perceived loudness across recordings. Recordings were then trimmed in *Audacity* to create stimuli of six different exposure durations: 25 ms, 50 ms, 100 ms, 200 ms, 400 ms, and 800 ms. For all recordings, a brief fade in and fade out of 12.5 ms was added to the beginning and end of the trimmed recordings to improve participants’ perceptual experience of the recordings.

The sustained vowels were chosen because the acoustic and thus voice-related information included is quasi steady state across the entire recording. Thus, any excerpt of the recording should be relatively comparable with any other excerpt from the same recording, which is a prerequisite for perceptual gating studies like the one implemented in this experiment (see also Mileva & Lavan, 2023).

Twenty-five milliseconds was chosen as the shortest duration because previous studies indicated that, for example, impressions of gender and dominance from voices (Mileva & Lavan, 2023; Owren et al., 2007) are already well established for voice recordings of a duration of 50 ms or less. Shorter durations were, however, not considered because they were unlikely to be perceivable as being human voices. The longest duration of 800 ms was chosen because previous trait perception studies have shown that many trait evaluations are well established by around 400 ms, suggesting that 800 ms of exposure should be sufficient to capture the emergence of initial impressions for person characteristics (McAleer et al., 2014; Mileva & Lavan, 2023; Owren et al., 2008; Purnell et al., 1999).

### Procedure

After reading an information sheet and providing consent, participants completed a sound check to confirm that sound playback was working adequately. During this sound check, participants were also able to adjust the playback volume to a comfortable level. After this, participants started the main experiment, in which they were asked to rate all 100 voices on 9-point scales for three of nine characteristics, the assignment of which was varied across participants. Characteristics were broadly grouped under three categories: physical characteristics (age, gender, health), trait characteristics (attractiveness, dominance, trustworthiness), and social characteristics (level of education, poshness, professionalism).

Selection of the physical and social characteristics was guided by the results of an experiment that collected free descriptions of people from voices (Lavan, 2023): The specific physical and social characteristics in the current experiment were among the most frequently mentioned types of person characteristics listed by lay listeners, suggesting that these characteristics are relevant to lay listeners when forming impressions of other people. The trait characteristics were selected on the basis of existing literature identifying attractiveness, dominance, and trustworthiness as being among the fundamental characteristics for trait perception (Lin et al., 2021; McAleer et al., 2014; Oosterhof & Todorov, 2008; Sutherland et al., 2013). Note that during stimulus selection, the 100 voices were sampled without directly manipulating any of the person characteristics rated except for gender, it was assumed that the sampled voices would differ sufficiently in terms of their perceived characteristics to result in a reasonable spread of responses in the rating tasks (described below) to support the statistical analyses.

**Fig. 1.**
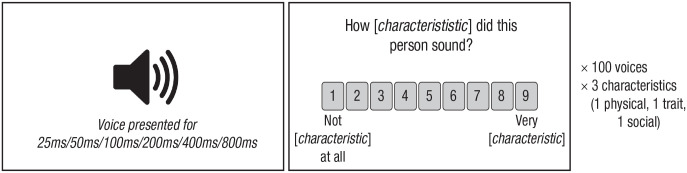
Illustration of the experimental task. On each trial, participants were presented with a voice, followed by a 9-point rating scale for a specific characteristic of that voice. Note that the instructions for the age and gender scales (not shown here) ran, respectively, from 1, *very masculine*, to 9, *very feminine*, and 1, a young adult, to 9, *a very old adult* (see Method for more details).

Each participant rated three of the nine characteristics. The assignment of rating tasks was counterbalanced across participants, such that each participant rated one physical characteristic, one trait characteristic, and one social characteristic. Rating tasks for the characteristics were presented in blocks. The order of blocks and stimuli within each block were randomized across participants. Exposure duration was manipulated between participants. Thus, each participant was assigned to rate voices for a single exposure duration (25 ms, 50 ms, 100 ms, 200 ms, 400 ms, or 800 ms) across the entire experiment (see Fig. 1). Thus, one participant might have rated all voices for age, dominance, and level of education with each voice across all blocks being presented for 100 ms, whereas another participant might have been asked to provide ratings for gender, attractiveness, and poshness on the basis of recordings of 800 ms for all blocks.

Before each block, participants were informed that they would be hearing 100 short voice recordings and that their task would be to rate the people in those recordings according to a specific characteristic (e.g., “How attractive is this person?”; 1, *not attractive at all*, to 9, *very attractive*). For age, the rating scale ran from 1, *a young adult*, to 9, *a very old adult*. For gender, the rating scale (“How masculine or feminine is this person?”) ran from 1, *very feminine*, and 9, *very masculine*. No further definitions of the different characteristics were provided for participants. Participants were asked to provide ratings quickly, following their gut reactions instead of overthinking their choices. They were furthermore informed that even though the recordings may be very short, they would not be able to replay any of them, such that they would need to listen attentively to the first (and only) presentation of each recording.

Each block also included nine vigilance trials, during which participants heard a voice generated via a text-to-speech synthesizer that instructed them to select a specific rating for the current trials instead of providing a judgment of the person. The vigilance trials thus functioned as an attention check as well as a check to confirm that participants were able to hear the recordings throughout the experiment.

### Data analysis

These ratings data were first analyzed by computing the interclass correlation coefficient (ICC2k; two-way random model, absolute agreement) alongside their 95% confidence intervals (CIs) as a measure of interrater agreement using the *psych* package in *R*. The ICC ranges between 0 (no agreement) and 1 (perfect agreement). In this study, high agreement indicated that listeners agreed with each other on which voices sounded similar on a given characteristic (e.g., more masculine vs. more feminine). I reasoned that if listeners agreed with each other, they must have tapped into some shared mental representation of the relevant person characteristic. High agreement was therefore taken as evidence that listeners indeed formed an impression of a person characteristic from a voice recording (see Mileva & Lavan, 2023). Conversely, low agreement was seen as evidence that likely no impression was formed and that participants provided noisy/random (or potentially idiosyncratic) ratings.

Although the ICC can shed light on how well different listeners agree with one another for each individual exposure duration, it cannot speak to how ratings of person characteristics relate to each other across different exposure durations. Following studies investigating the time course of trait perception from faces and voices (e.g., Mileva & Lavan, 2023; Todorov et al., 2009; Willis & Todorov, 2006), I therefore also analyzed the data by correlating mean ratings per voice recording from each of the shorter exposure durations (25 ms, 50 ms, 100 ms, 200 ms, 400 ms) with the longest exposure duration (800 ms). Alongside the correlation coefficient, 95% CIs were computed. The longest exposure duration was thus used as a benchmark, after which I expected that initial impressions had been formed.

In conjunction with the ICC, correlations between the shorter and longest exposure durations can then be used as means of tracking the formation of impressions across time: For example, if the ICC is high for ratings of age after 50 ms of exposure as well as 800 ms of exposure, this would indicate that the impressions of age formed after 50 ms and 800 ms are likely based on similar information. However, if the correlation coefficient was low in the presence of high ICCs, it would indicate that although listeners were able to form a shared impression, additional information became available with increasing exposure and influenced the impressions, thus systematically changing them. The lower the ICCs, the less reliable and informative the mean ratings for voice recordings, such that in the context, correlation coefficients should also be lower, although mean ratings derived from data with low ICCs should be interpreted with caution.

All analyses were conducted across all 100 voices as well as for the 50 female and 50 male voices separately. This was done to take into account previous research into trait perception showing that some traits that were investigated in this study show gender differences, such that, for example, male voices are usually associated with being more dominant than female voices (e.g., Lavan et al., 2021; McAleer et al., 2014). Such gender effects could inflate correlations and measures of agreement.

Broad differences and trends in the ICCs and correlations within and across exposure times and characteristics are interpreted in light of the 95% CIs computed for both measures. Where CIs do not overlap—across traits and/or across time points—values are considered to be different from one another. This is a relatively conservative threshold because CIs that do not overlap but touch are considered to be similar to an α level of *p* = .01 (Cumming, 2009). For completeness, correlations between item-wise mean ratings for all characteristics and all exposure durations are plotted in Figure S1 in the Supplemental Material.

## Results

### Listeners formed initial impressions of person characteristics by 400 ms

**Fig. 2.**
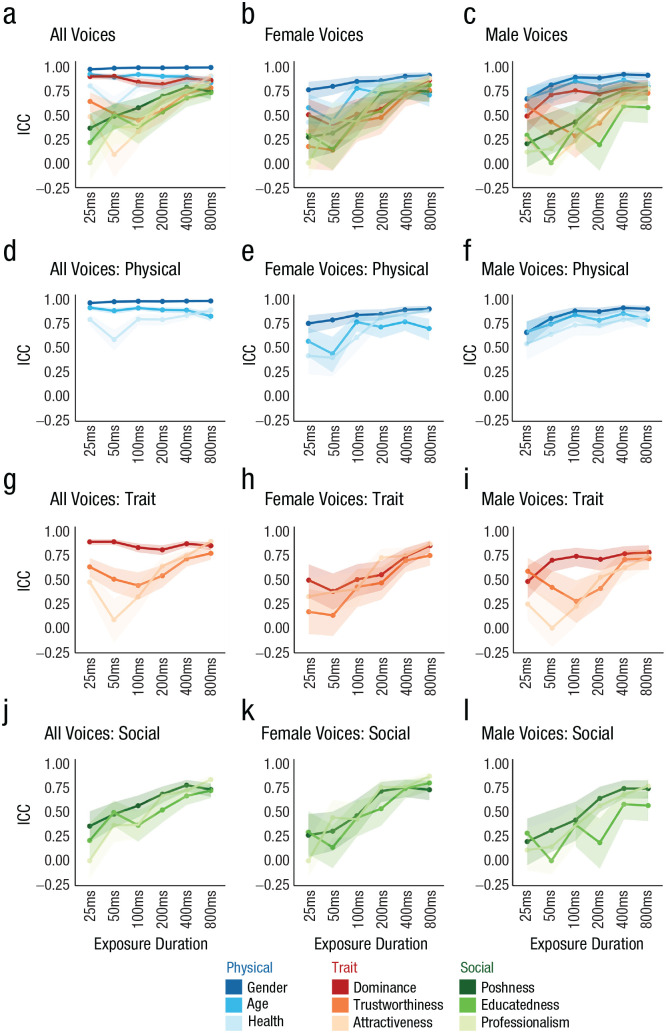
Intraclass correlation coefficients (ICCs) for ratings of each person characteristic at each exposure duration, separately for all voices (left column), female voices only (middle column), and male voices only (right column). The top row (a–c) shows results for gender. The second to fourth rows show ICCs for, respectively, physical characteristics (d–f), trait characteristics (g–i), and social characteristics (j–l). Error bands represent 95% confidence intervals. Analysis S2 in the Supplemental Material available online reproduces all values plotted in this figure.

After 400 ms of exposure to a voice, the ICCs were above .67 for all nine person characteristics and above .73 after 800 ms of exposure (Fig. 2a). A very similar picture emerged when female and male voices were analyzed separately: After 400 ms of exposure, ICCs for female voices exceeded .70 and ICCs for male voices were above .63 (Figs. 2b and 2c; after 800 ms of exposure, ICCs > .70 for female voices and ICCs > .57 for male voices). Taken together, the substantial agreement across listeners shows that for all nine characteristics, listeners had formed initial impressions based on some shared representations after only 400 ms of exposure to the voice.

Notably, however, despite the overall good agreement after 400 ms of exposure, the agreement for several characteristics (e.g., educatedness, attractiveness) still increased between 400 ms and 800 ms of exposure. This indicates that although impressions had largely been formed, moderate changes or updates to these impressions still occurred, even after 400 ms of exposure to a relatively steady-state vowel.

**Fig. 3.**
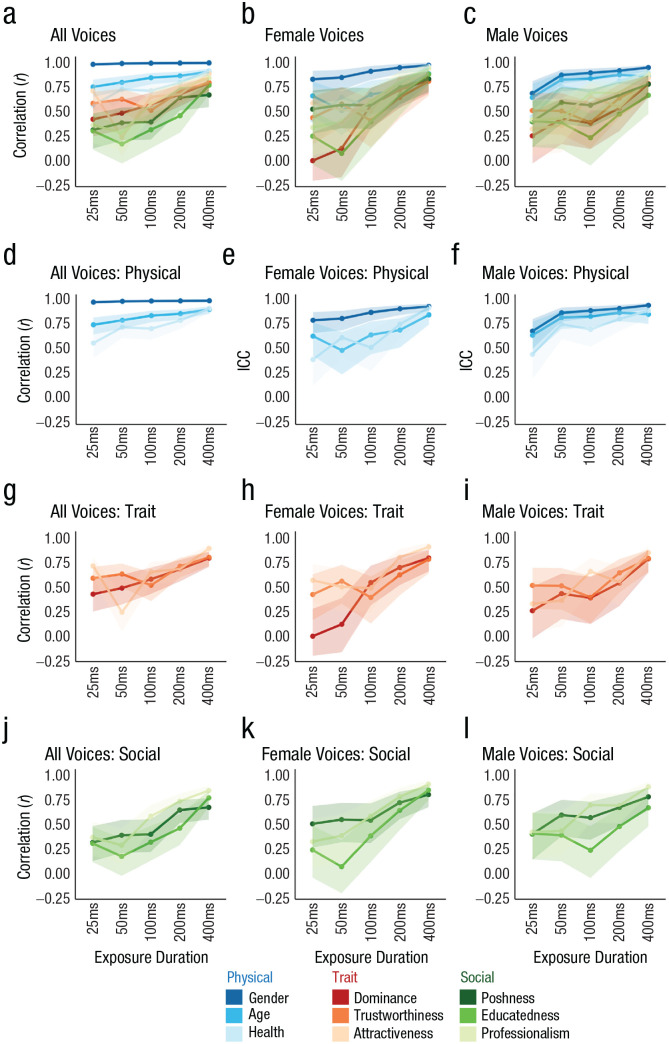
Correlation coefficients for ratings of each person characteristic at each exposure duration, separately for all voices (left column), female voices only (middle column), and male voices only (right column). The top row (a–c) shows results for gender. The second to fourth rows show correlations for, respectively, physical characteristics (d–f), trait characteristics (g–i), and social characteristics (j–l). Error bands represent 95% confidence intervals. Analysis S2 in the Supplemental Material available online reproduces all values plotted in this figure.

The correlation coefficients between mean ratings of voices following 400 ms and 800 ms of exposure were generally high (> .67) when all voices were examined (Fig. 3a). Similarly, for female voices, correlations between the mean ratings of recordings of 400 ms and 800 ms in duration exceeded .77, and for male voices, correlation coefficients exceeded .66 (Figs. 3b and 3c). This shows that the impressions formed after 400 ms and 800 ms of exposure, respectively, were not only shared across listeners within a certain exposure duration but also relatively similar across these two exposure durations, as indicated by the strong correlations observed for each of the characteristics, within and across gender.

This is in line with previous studies that have established that listeners can form impressions of character traits rapidly within approximately 400 ms depending on the specific trait (Baus et al., 2019; McAleer et al., 2014; Mileva & Lavan, 2023). Similarly, the accuracy of judgments on speaker sex/gender has also been shown to emerge very quickly in under 15 ms (Owren et al., 2007). The current study extends these findings and underlines that listeners can rapidly form an initial impression of a person on any number of characteristics (physical, trait, social). Although the objective accuracy of these impressions may vary dramatically, subjective impressions as measured in this study appear to be informed by shared stereotypes.

### The time course of initial impression formation differs for different person characteristics

When looking at how ICCs evolve across the different exposure durations, it becomes apparent that the time courses over which initial impressions of person characteristics emerge differ substantially across the different characteristics. Across male and female voices, Figure 2 shows that impressions of physical characteristics (age, gender, health; Figs. 2d–2f) were already well established after only 25 ms, as indicated by relatively flat time courses. For trait characteristics (attractiveness, dominance, trustworthiness; Figs. 2g–2i) and social characteristics (educatedness, poshness, professionalism; Figs. 2j–2l), impressions were mostly formed on a somewhat slower time scale—with the notable exception of dominance impressions—as indicated by the gradually rising trajectories. When male and female voices were examined separately, similar trends emerged in the presence of generally lower levels of agreement as well as some gender-specific patterns (cf. dominance). These broad trends are described in more detail below.

#### All voices

Impressions of physical characteristics (age, gender, health) had overall high ICCs across all exposure durations and were therefore already well formed from the shortest exposure (25 ms) to the longest exposure (800 ms). This flat trajectory marks the time course of physical impressions as quite different from the time course of most of the remaining social and trait impressions (except for dominance). This claim is supported by the fact that the CIs for age and gender perception did not overlap with the CIs for social and trait characteristics until 800 ms of exposure (but see dominance). Similarly, the CIs for health impressions started to overlap with the CIs for some social and trait impressions only after 200 ms of exposure.

When looking at the overall level of agreement for the three physical characteristics was examined, it was apparent that agreement was highest for gender impressions, followed closely by age impressions and, finally, health impressions. This graded nature of the global agreement can be explained in several ways: For example, the overall lower agreement for health may indicate that listeners’ representations of healthy- versus unhealthy-sounding voices may have been somewhat less well formed and thus somewhat fuzzier compared with, for example, representations of male- versus female-sounding voices. Such fuzzier representations could have introduced some noise in listeners’ ratings, thus also potentially explaining the globally lower ICC. Alternatively, listeners may have had equally well-formed representations for health versus gender impressions from voices, but there may have been more idiosyncratic aspects in listeners’ representation of what a healthy versus an unhealthy voice may sound like. Despite these moderate global differences in agreement for these impressions, the data nonetheless suggest that impressions of all three physical characteristics were already well established by 25 ms and changed relatively little across exposure durations.

Among the three trait characteristics (attractiveness, dominance, trustworthiness), the nature of the time courses was more varied compared to those for the physical characteristics described above. Across all voices, impressions of dominance were established quickly after only 25 ms, with ICCs being consistently high (> .81) across all exposure durations. Meanwhile, impressions of attractiveness and trustworthiness were formed more slowly, starting with moderate agreement, indicated by ICCs of .48 for attractiveness and .64 for trustworthiness after 25 ms of exposure and rising—with some notable fluctuations—to good agreement, with ICCs of .90 and .78, respectively, after 800 ms of exposure (see also McAleer et al., 2014). Impressions of trustworthiness and attractiveness thus generally followed a more gradual time course. As a result, the relatively rapid and stable time course of dominance impressions appears to be different from the remaining trait impressions and seems to more closely resemble the flat time courses of the physical characteristics. In fact, the 95% CIs for dominance overlap with age and health impressions but did not overlap with the remaining trait and social characteristics until after 800 ms of exposure. This faster formation of impressions of dominance versus other trait characteristics has also been reported previously (Mileva & Lavan, 2023). Similarly, this observation may align with reports that male-sounding voices are usually rated substantially higher in dominance compared with female-sounding voices (Lavan et al., 2021; McAleer et al., 2014; see Fig. S2 in the Supplemental Material): If categorical gender differences in dominance ratings exist and impressions of gender are formed rapidly from voices, agreement for dominance ratings should be inflated by these gender differences when agreement across male and female voices is examined. This issue will be addressed below (and partially confirmed) in discussions of analyses conducted separately for male and female voices.

Finally, the time courses of all three social characteristics (educatedness, poshness, professionalism) were similar to one another, beginning with relatively low ICCs (< .37) after 25 ms of exposure, increasing relatively steadily with increasing exposure (to ICCs > .73) after 800 ms of exposure. Descriptively speaking, the ICCs for all social characteristics were the lowest out of all nine characteristics at only 25 ms of exposure. However, with CIs overlapping with some trait characteristics at 25 ms and all other exposure durations, the time courses for social characteristics can be considered as following a gradual time course that is similar to that of the trait characteristics of trustworthiness and attractiveness.

#### Male versus female voices

When ICCs for male and female voices were examined separately, a similar picture emerged for male voices as was observed across all voices (Figs. 2b and 2c). Agreement for impressions of physical characteristics and dominance were formed quickest. Notably, however, ICCs were generally somewhat lower for exposure durations under 100 ms compared with ICCs computed across all voices, potentially indicating that the inclusion of both genders—and thus a more varied voice sample—may have supported the very rapid formation of impressions for these characteristics observed across all voices. Impressions for the remaining trait and social characteristics emerge more gradually and are comparable with the time courses observed across all voices: ICCs for the shorter exposure durations were low, increasing toward the good to high agreement observed after 400 ms and 800 ms of exposure.

For female voices, a slightly different picture emerged: Impressions of speaker gender again formed rapidly in under 100 ms, with agreement being high throughout (ICCs > .75). For the two remaining physical characteristics, health and age, ICCs after 25 ms and 50 ms of exposure were numerically higher than most trait and social characteristics. However, agreement was initially only moderate but then rose higher after 400 ms and 800 ms of exposure (ICCs > .71), suggesting a somewhat slower trajectory for impression formation. Strikingly, impressions of dominance for female voices only were not established rapidly as observed for male voices but seem to have behaved like the other trait and social characteristics, showing gradually increasing agreement over time. This may suggest an overall potentially somewhat slower time course of impression formation from female voices. These moderate gender differences align with reports in the literature, showing that listeners have been shown to perceive masculinity in the voice more quickly and accurately than femininity (Coleman, 1971; Lass et al., 1976; Owren et al., 2007). Similarly, Mileva and Lavan (2023) report gender differences in the speed of trait impressions, showing a slower time course for dominance and attractiveness for female voices. For line graphs directly contrasting time courses for male versus female voices, see Analysis S3 in the Supplemental Material.

### Impressions formed after shorter exposures often resemble impressions formed from longer exposures

#### All voices

The second measure of interest, correlations between mean ratings of voices for the shorter exposure durations (25 ms–400 ms) and the longest exposure duration (800 ms), showed broadly similar patterns of results as observed for the ICC (see Fig. 3). Across all voices, the highest correlations occurred for all three physical characteristics across the different exposure durations. This shows not only that impressions were formed rapidly in terms of interrater agreement but also that the impressions that were formed from shorter exposures were also similar to those formed after the longest exposures. Correlations for physical properties were again highest for speaker gender (*r*s = .98–1.00), followed by age (*r*s = .65–.87) and health (*r*s = .56–.92; Fig. 3d).

For trait characteristics, correlations were generally numerically lower compared with physical characteristics across the different exposure duration (*r*s = .24–.89, Figs. 3g–3i). Intriguingly, correlation strengths for dominance were well aligned with correlation strengths for attractiveness and trustworthiness and did not, for example, stand out as being high throughout, as was the case for agreement. This finding suggests that although listeners clearly agreed on which voices were more or less dominant for each of the individual exposure durations, as measured by the ICC, the impressions evolved to some degree over time with increasing exposure durations to gradually become more similar to the impressions after 800 ms of exposure.

Finally, for social characteristics, correlations were numerically the weakest for the shorter exposure durations, across all voices. However, much like for the ICCs, the correlations for social characteristics were broadly in line with correlation strengths for trait properties (*r*s = .31–.84, Figs. 3j–3l), as indicated by often overlapping CIs.

#### Male versus female voices

When correlations for male and female voices were examined separately, a very similar picture to that observed across all voices emerged again for male voices (Fig. 2f). Correlations were strongest for physical characteristics, followed by trait and social characteristics, which were in turn very similar to one another in correlation strength. As was already observed for correlations for all voices, dominance was again aligned with the remaining trait and social characteristics instead.

For female voices, as was already seen for the ICC, only gender stood out as having relatively strong correlations across all exposure durations (Fig. 2e). The remaining physical, trait, and social characteristics evolved in broadly similar ways. Dominance impressions for female voices furthermore showed no clear correlation between 25 ms to 50 ms and 800 ms of exposure.

Overall, these analyses confirm that impressions that are formed from shorter exposure durations often bear substantial resemblance to the impressions formed after 800 ms of exposure. There are, however, some notable exceptions: For example, impressions of dominance appear to evolve over time, as indicated by increasing correlation coefficients, although the interrater agreement was almost uniformly high across all exposure durations (for male and across all voices). It should again be noted that correlations for exposure durations associated with low agreement need to be interpreted with caution because low agreement likely results in unreliable item-wise means (see also Mileva & Lavan, 2023).

## General Discussion

The current study shows that different person characteristics are associated with different time courses during impression formation. Initial impressions of physical characteristics were formed within 25 ms of exposure, whereas initial impressions formation for trait and social characteristics generally required several hundred milliseconds of exposure to a voice—except for impressions of dominance. Thus, the perception of physical characteristics appears to at times differ from the perception of social and trait characteristics.

One way in which these differential time courses can be explained is related to the acoustic cues underpinning the perception of physical characteristics versus social characteristics. On the one hand, physical characteristics may be more easily differentiated on the basis of acoustic cues than social and trait characteristics. For example, human voices are sexually dimorphic: Male voices are generally substantially lower in pitch than female voices, making it easy to distinguish between the two types of voices. If listeners use pitch as an acoustic shorthand for gender judgments, this could explain the very fast time course of impression formation for gender in the current study (Owren et al., 2007). Similarly, it could be argued that the most salient acoustic cues to social characteristics may not be encoded in the sound of the voice (as sampled here via sustained vowels) but may be primarily found in *how* someone speaks (e.g., accent perception; see Bayard et al., 2001; Purnell et al., 1999; Jiang et al., 2020; Mauchand & Pell, 2022a, 2022b), which inevitably unfolds over a longer time scale, that was not sampled here. Such a reduction of salient acoustic cues in the current study could explain the slower time course of the perception of social characteristics.

However, although an explanation that depends on the clarity and availability of acoustic cues can explain some of these findings, there are indications that such an explanation alone is too simplistic: For example, although sustained vowels may indeed not include all salient cues to social characteristics, the agreement for social characteristics was nonetheless high after more than 400 ms of exposure. This means that some cues must have been available in the sound of the voice to result in high agreement. Similarly, although the sexual dimorphism of human voices may explain the rapid impression formation for gender perception, trait impressions have also been shown to be linked to perceived pitch (e.g., McAleer et al., 2014), which could have in principle supported rapid impression formation for traits. Acoustic cues can thus not offer a comprehensive explanation on their own.

In the current study, the perception of physical characteristics, such as gender, health, and age, alongside dominance in males were perceived most rapidly out of all characteristics examined. It is notable that the perception of these specific characteristics is often studied in light of sexual selection and formidability perception in the context of comparative and evolutionary psychology (e.g., Aung et al., 2021; Puts et al., 2012). These findings could thus hint at physical characteristics and dominance being processed in a different way as or with different perceptual aims than the remaining characteristics that were processed somewhat more slowly—on the basis of different evolutionary origins. This explanation is of course highly speculative, and the current data cannot directly speak to whether there are evolutionary differences in processing different kinds of person characteristics. Engaging in such speculations from multiple perspectives around the processes that may explain the current results, however, highlights that it is unlikely that there is a single explanation. Instead, it seems more likely that different mechanisms driven by interactions of acoustic cues and perceptual processes are at work to support person perception from voices.

Finding evidence for staggered time courses tracking the emergence of initial impressions of person characteristics from voices confirms that there may be complexities to person perception from voices that warrant further exploration: For example, with some of these person characteristics being perceived more rapidly than others, there is a possibility that there may be a temporal or perceptual hierarchy for person perception from voices. Therefore, the rapidly perceived physical characteristics influence the perception of the slower impressions in a bottom-up fashion. The presence of such a hierarchy and possible interactions between different person characteristics may thus also explain more generally how overgeneralization effects of perceived gender, age, and beyond on trait or person perception may arise (Zebrowitz, 2011). For example, the rapid perception of a voice being female may restrict the relatively slower impressions of that person’s trustworthiness to being relatively high (Schirmer et al., 2020; see also Lavan & McGettigan, 2023).

## Limitations and Future Work

Although the existence of a perceptual hierarchy during person perception would help explain some previously reported effects, many questions arising from such a hierarchy remain unanswered: For example, how many levels are there in this proposed hierarchy and how would they differ from one another (e.g., Ochs, 1992, and Silverstein, 2003, from sociolinguistic perspectives on hierarchies during person perception)? For the current results, where physical characteristics were processed most quickly, would that mean that these characteristics are indeed processed at a different “level” than other characteristics investigated? Similarly, although staggered time courses introduce the possibility of bottom-up influences of faster impressions on slower ones, there will likely also be various top-down processes interacting with different impressions before a listener arrives at their first, perhaps holistic, impression of the person they are hearing. One example of such top-down influences would be a situation where we are expecting to hear the voice of a child but we realise that we were, in fact, listening to an adult woman’s voice with a high pitch. Would the expectation of hearing a child voice have influenced what we thought of the voice and when this happened? Finally, the current study asked only how and when impressions of other people are formed based on short vowels that included minimal vocal information. These early impressions that are based on the sound of the voice are unlikely to remain unchanged as additional information from linguistic content and situational context becomes available to listeners in real-life social interactions. How then do impressions evolve beyond the 800-ms window investigated here over the course of a first encounter with another person, where additional information can be perceived that may feed into initial impressions? Beyond considerations of how the current results may (or may not) generalize well to more naturalistic voice stimuli and social settings, additional questions arise about what the data from behavioral experiments such as this one tap into: In a strict view, the results can speak only to the fact that a certain amount of exposure to a voice (plus the reaction time) was sufficient for listeners to explicitly judge specific person characteristics. This is likely a somewhat imprecise measure. How therefore does this measure relate to true initial impression formation? These questions and considerations arising from the current study highlight that much more work, using a variety of stimuli, tasks, and methods, is required to examine how complex and multivariate impressions of people arise from their voices. This is an exciting prospect. I argue that it will be essential for future work to take a broader view of person perception from voices and to investigate how multiple aspects of person perception interact within the same experiments to be able to begin to tackle these questions.

## Supplemental Material

sj-docx-1-pss-10.1177_09567976231161565 – Supplemental material for The Time Course of Person Perception From Voices: A Behavioral Study Supplemental material, sj-docx-1-pss-10.1177_09567976231161565 for The Time Course of Person Perception From Voices: A Behavioral Study by Nadine Lavan in Psychological Science

## Transparency

*Action Editor:* Lasana Harris

*Editor:* Patricia J. Bauer

*Author Contributions*

**Nadine Lavan:** Conceptualization; Data curation; Formal analysis; Funding acquisition; Investigation; Methodology; Project administration; Resources; Writing – original draft.

## References

[bibr1-09567976231161565] Aung T. Goetz S. Adams J. McKenna C. Hess C. Roytman S. Cheng J. T. Zilioli S. Puts D. (2021). Low fundamental and formant frequencies predict fighting ability among male mixed martial arts fighters. Scientific Reports, 11, Article 905. 10.1038/s41598-020-79408-6 PMC7806622 33441596

[bibr2-09567976231161565] Baus C. McAleer P. Marcoux K. Belin P. Costa A. (2019). Forming social impressions from voices in native and foreign languages. Scientific Reports, 9, Article 414. 10.1038/s41598-018-36518-6 PMC6344506 30674913

[bibr3-09567976231161565] Bayard D. Weatherall A. Gallois C. Pittam J. (2001). Pax Americana? Accent attitudinal evaluations in New Zealand, Australia and America. Journal of Sociolinguistics, 5(1), 22–49.

[bibr4-09567976231161565] Boersma P. Weenink D. (2020). Praat: Doing phonetics by computer (Version 6.1.26) [Computer software]. https://www.fon.hum.uva.nl/praat/

[bibr5-09567976231161565] Caballero J. A. Mauchand M. Jiang X. Pell M. D. (2021). Cortical processing of speaker politeness: Tracking the dynamic effects of voice tone and politeness markers. Social Neuroscience, 16(4), 423–438. 34102955 10.1080/17470919.2021.1938667

[bibr6-09567976231161565] Coleman R. O. (1971). Male and female voice quality and its relationship to vowel formant frequencies. Journal of Speech and Hearing Research, 14(3), 565–577. 5163891 10.1044/jshr.1403.565

[bibr7-09567976231161565] Cumming G. (2009). Inference by eye: Reading the overlap of independent confidence intervals. Statistics in Medicine, 28(2), 205–220. 18991332 10.1002/sim.3471

[bibr8-09567976231161565] D’Onofrio A. (2020). Personae in sociolinguistic variation. Wiley Interdisciplinary Reviews: Cognitive Science, 11(6), Article e1543. 10.1002/wcs.1543 32914575

[bibr9-09567976231161565] Groyecka-Bernard A. Pisanski K. Frackowiak T. Kobylarek A. Kupczyk Oleszkiewicz A. Sabiniewicz A. Wróbel M. Sorokowski P. (2022). Do voice-based judgments of socially relevant speaker traits differ across speech types. Journal of Speech, Language, and Hearing Research, 65(10), 3674–3694. 10.1044/2022_JSLHR-21-00690 36167068

[bibr10-09567976231161565] Jiang X. Gossack-Keenan K. Pell M. (2020). To believe or not to believe? How voice and accent information in speech alter listener impressions of trust. Quarterly Journal of Experimental Psychology, 73(1), 55–79. 10.1177/1747021819865833 31293191

[bibr11-09567976231161565] Jiang X. Pell M. D. (2015). On how the brain decodes vocal cues about speaker confidence. Cortex, 66, 9–34. 25796072 10.1016/j.cortex.2015.02.002

[bibr12-09567976231161565] Jiang X. Pell M. D. (2016a). The feeling of another’s knowing: How “mixed messages” in speech are reconciled. Journal of Experimental Psychology: Human Perception and Performance, 42(9), 1412–1428. 27123678 10.1037/xhp0000240

[bibr13-09567976231161565] Jiang X. Pell M. D. (2016b). Neural responses towards a speaker’s feeling of (un)knowing. Neuropsychologia, 81, 79–93. 26700458 10.1016/j.neuropsychologia.2015.12.008

[bibr14-09567976231161565] Lass N. J. Hughes K. R. Bowyer M. D. Waters L. T. Bourne V. T. (1976). Speaker sex identification from voiced, whispered, and filtered isolated vowels. The Journal of the Acoustical Society of America, 59(3), 675–678. 1254794 10.1121/1.380917

[bibr15-09567976231161565] Latinus M. Taylor M. J. (2012). Discriminating male and female voices: Differentiating pitch and gender. Brain Topography, 25(2), 194–204. 22080221 10.1007/s10548-011-0207-9

[bibr16-09567976231161565] Lavan N. (2023). How do we describe other people from voices and faces? Cognition, 230, Article 105253. 10.1016/j.cognition.2022.105253 PMC7618126 36215763

[bibr17-09567976231161565] Lavan N. McGettigan C. (2023). A model of person perception from voices. PsyArXiv. 10.31234/osf.io/shxa6 PMC11041786 38665246

[bibr18-09567976231161565] Lavan N. Mileva M. Burton A. M. Young A. W. McGettigan C. (2021). Trait evaluations of faces and voices: Comparing within- and between-person variability. Journal of Experimental Psychology: General, 150(9), 1854–1869. 33734774 10.1037/xge0001019 PMC7612101

[bibr19-09567976231161565] Lin C. Keles U. Adolphs R. (2021). Four dimensions characterize attributions from faces using a representative set of English trait words. Nature Communications, 12, Article 5168. 10.1038/s41467-021-25500-y PMC8397784 34453054

[bibr20-09567976231161565] Mahrholz G. Belin P. McAleer P. (2018). Judgements of a speaker’s personality are correlated across differing content and stimulus type. PLOS ONE, 13(10), Article e0204991. 10.1371/journal.pone.0204991 PMC6171871 30286148

[bibr21-09567976231161565] Mauchand M. Pell M. D. (2022a). French or Québécois? How speaker accents shape implicit and explicit intergroup attitudes among francophones in Montréal. Canadian Journal of Behavioural Science / Revue Canadienne Des Sciences Du Comportement, 54(1), 1–8.

[bibr22-09567976231161565] Mauchand M. Pell M. D. (2022b). Listen to my feelings! How prosody and accent drive the empathic relevance of complaining speech. Neuropsychologia, 175, Article 108356. 10.1016/j.neuropsychologia.2022.108356 36037914

[bibr23-09567976231161565] McAleer P. Todorov A. Belin P. (2014). How do you say ‘hello’? Personality impressions from brief novel voices. PLOS ONE, 9(3), Article e90779. 10.1371/journal.pone.0090779 PMC3951273 24622283

[bibr24-09567976231161565] Mileva M. Lavan N. (2023). Trait impressions from voices are formed rapidly within 400 ms of exposure. Journal of Experimental Psychology: General. Advance online publication. 10.1037/xge0001325 36745087

[bibr25-09567976231161565] Moyse E. (2014). Age estimation from faces and voices: A review. Psychologica Belgica, 54(3), 255–265.

[bibr26-09567976231161565] Ochs E. (1992). Indexing gender. In Duranti A. Goodwin C. (Eds.), Rethinking context: Language as an interactive phenomenon (pp. 335–358). Cambridge University Press.

[bibr27-09567976231161565] Oosterhof N. N. Todorov A. (2008). The functional basis of face evaluation. Proceedings of the National Academy of Sciences, USA, 105(32), 11087–11092. 10.1073/pnas.0805664105 PMC2516255 18685089

[bibr28-09567976231161565] Owren M. J. Berkowitz M. Bachorowski J. A. (2007). Listeners judge talker sex more efficiently from male than from female vowels. Perception & Psychophysics, 69(6), 930–941. 18018974 10.3758/bf03193930

[bibr29-09567976231161565] Purnell T. Idsardi W. Baugh J. (1999). Perceptual and phonetic experiments on American English dialect identification. Journal of Language and Social Psychology, 18(1), 10–30.

[bibr30-09567976231161565] Puts D. A. Apicella C. L. Cárdenas R. A. (2012). Masculine voices signal men’s threat potential in forager and industrial societies. Proceedings of the Royal Society B: Biological Sciences, 279(1728), 601–609. 10.1098/rspb.2011.0829 PMC3234546 21752821

[bibr31-09567976231161565] Pützer M. Barry W. J. (n.d.). Saarbrücker voice database. http://stimmdb.coli.uni-saarland.de/

[bibr32-09567976231161565] Rendall D. Vokey J. R. Nemeth C. (2007). Lifting the curtain on the Wizard of Oz: Biased voice-based impressions of speaker size. Journal of Experimental Psychology: Human Perception and Performance, 33(5), 1208–1219. 17924818 10.1037/0096-1523.33.5.1208

[bibr33-09567976231161565] Rezlescu C. Penton T. Walsh V. Tsujimura H. Scott S. K. Banissy M. J. (2015). Dominant voices and attractive faces: The contribution of visual and auditory information to integrated person impressions. Journal of Nonverbal Behavior, 39(4), 355–370.

[bibr34-09567976231161565] Schirmer A. Chiu M. H. Lo C. Feng Y.-J. Penney T. B. (2020). Angry, old, male – and trustworthy? How expressive and person voice characteristics shape listener trust. PLOS ONE, 15(5), Article e0232431. 10.1371/journal.pone.0232431 PMC7197804 32365066

[bibr35-09567976231161565] Silverstein M. (2003). Indexical order and the dialectics of sociolinguistic life. Language & Communication, 23(3–4), 193–229.

[bibr36-09567976231161565] Sutherland C. A. Oldmeadow J. A. Santos I. M. Towler J. Burt D. M. Young A. W. (2013). Social inferences from faces: Ambient images generate a three-dimensional model. Cognition, 127(1), 105–118. 23376296 10.1016/j.cognition.2012.12.001

[bibr37-09567976231161565] Todorov A. Pakrashi M. Oosterhof N. N. (2009). Evaluating faces on trustworthiness after minimal time exposure. Social Cognition, 27(6), 813–833.

[bibr38-09567976231161565] Vergis N. Jiang X. Pell M. D. (2020). Neural responses to interpersonal requests: Effects of imposition and vocally-expressed stance. Brain Research, 1740, Article 146855. 10.1016/j.brainres.2020.146855 32348774

[bibr39-09567976231161565] Willis J. Todorov A. (2006). First impressions: Making up your mind after a 100-ms exposure to a face. Psychological Science, 17(7), 592–598. 16866745 10.1111/j.1467-9280.2006.01750.x

[bibr40-09567976231161565] Zebrowitz L. A. (2011). Ecological and social approaches to face perception. In Calder A. J. Rhodes G. Johnson M. H. Haxby J. V. (Eds.), Oxford handbook of face perception (pp. 31–50). Oxford University Press.

