# Supplementary material for: The Time Course of Person Perception From Voices: A Behavioral Study
Source: Psychol Sci. 2023 May 25;34(7):771–83. doi: 10.1177/09567976231161565 (PMC13032837; doi:10.1177/09567976231161565)

**Supplementary Methods**

*Supplementary Table 1: Participant numbers per condition and exposure duration.*

| **Exposure** | **Age** | **Health** | **Gender** | **Attractiveness** | **Dominance** | **Trustworthiness** | **Educatedness** | **Poshness** | **Professionalism** |
| --- | --- | --- | --- | --- | --- | --- | --- | --- | --- |
| 25 | 32 | 32 | 33 | 32 | 32 | 33 | 32 | 33 | 32 |
| 50 | 32 | 34 | 34 | 34 | 32 | 22 | 32 | 22 | 34 |
| 100 | 33 | 31 | 32 | 31 | 33 | 32 | 33 | 32 | 31 |
| 200 | 31 | 35 | 33 | 35 | 31 | 33 | 31 | 33 | 35 |
| 400 | 35 | 36 | 33 | 36 | 35 | 33 | 35 | 33 | 36 |
| 800 | 33 | 34 | 32 | 34 | 33 | 32 | 33 | 32 | 34 |

**Supplementary Analysis 1**

Below tables show all correlation coefficients and ICCs are reproduced.

*Supplementary Table 2: Tables showing the correlation coefficients associated with Figure 2 in the main manuscript. Correlation coefficients and 95% CIs are provided across all voices and for male and female voices respectively.*

| **Physical Characteristic** | **Exposure** | **ICC** | **95% CIs** | **F** | **95% CIs** | **M** | **95% CIs** |
| --- | --- | --- | --- | --- | --- | --- | --- |
| Gender | 25 | 0.97 | [0.97;0.98] | 0.76 | [0.68;0.83] | 0.66 | [0.55;0.76] |
|  | 50 | 0.99 | [0.98;0.99] | 0.80 | [0.72;0.86] | 0.81 | [0.74;0.87] |
|  | 100 | 0.99 | [0.99;0.99] | 0.85 | [0.79;0.89] | 0.89 | [0.85;0.92] |
|  | 200 | 0.99 | [0.99;0.99] | 0.86 | [0.80;0.90] | 0.88 | [0.84;0.92] |
|  | 400 | 0.99 | [0.99;0.99] | 0.90 | [0.87;0.93] | 0.92 | [0.89;0.95] |
|  | 800 | 0.99 | [0.99;1.00] | 0.91 | [0.88;0.94] | 0.91 | [0.88;0.94] |
| Age | 25 | 0.92 | [0.90;0.94] | 0.57 | [0.44;0.70] | 0.67 | [0.56;0.77] |
|  | 50 | 0.89 | [0.86;0.92] | 0.44 | [0.28;0.60] | 0.75 | [0.67;0.83] |
|  | 100 | 0.92 | [0.90;0.94] | 0.78 | [0.70;0.84] | 0.85 | [0.79;0.90] |
|  | 200 | 0.90 | [0.88;0.92] | 0.72 | [0.63;0.81] | 0.79 | [0.72;0.86] |
|  | 400 | 0.90 | [0.87;0.92] | 0.78 | [0.70;0.85] | 0.86 | [0.81;0.90] |
|  | 800 | 0.83 | [0.79;0.87] | 0.71 | [0.61;0.79] | 0.80 | [0.73;0.86] |
| Health | 25 | 0.80 | [0.75;0.85] | 0.42 | [0.26;0.58] | 0.55 | [0.41;0.67] |
|  | 50 | 0.59 | [0.50;0.68] | 0.40 | [0.24;0.56] | 0.65 | [0.53;0.75] |
|  | 100 | 0.80 | [0.75;0.85] | 0.61 | [0.49;0.72] | 0.74 | [0.65;0.82] |
|  | 200 | 0.80 | [0.75;0.85] | 0.82 | [0.76;0.88] | 0.74 | [0.65;0.82] |
|  | 400 | 0.84 | [0.80;0.88] | 0.85 | [0.80;0.90] | 0.80 | [0.73;0.86] |
|  | 800 | 0.90 | [0.87;0.92] | 0.90 | [0.86;0.93] | 0.83 | [0.76;0.88] |

| **Trait Characteristic** | **Exposure** | **ICC** | **95% CIs** | **F** | **95% CIs** | **M** | **95% CIs** |
| --- | --- | --- | --- | --- | --- | --- | --- |
| Attractiveness | 25 | 0.48 | [0.37;0.58] | 0.33 | [0.19;0.48] | 0.25 | [0.11;0.41] |
|  | 50 | 0.09 | [-0.11;0.27] | 0.38 | [0.20;0.54] | 0.00 | [-0.23;0.24] |
|  | 100 | 0.33 | [0.19;0.46] | 0.41 | [0.26;0.55] | 0.23 | [0.06;0.40] |
|  | 200 | 0.65 | [0.56;0.72] | 0.74 | [0.65;0.81] | 0.53 | [0.39;0.66] |
|  | 400 | 0.75 | [0.69;0.81] | 0.76 | [0.67;0.83] | 0.63 | [0.51;0.74] |
|  | 800 | 0.90 | [0.88;0.93] | 0.88 | [0.83;0.92] | 0.75 | [0.66;0.82] |
| Dominance | 25 | 0.90 | [0.87;0.92] | 0.50 | [0.35;0.64] | 0.49 | [0.34;0.63] |
|  | 50 | 0.90 | [0.88;0.92] | 0.38 | [0.22;0.54] | 0.71 | [0.61;0.80] |
|  | 100 | 0.84 | [0.80;0.88] | 0.51 | [0.36;0.64] | 0.75 | [0.66;0.83] |
|  | 200 | 0.82 | [0.77;0.86] | 0.56 | [0.42;0.68] | 0.72 | [0.62;0.80] |
|  | 400 | 0.88 | [0.85;0.91] | 0.75 | [0.66;0.82] | 0.78 | [0.70;0.84] |
|  | 800 | 0.86 | [0.82;0.89] | 0.86 | [0.81;0.90] | 0.79 | [0.72;0.85] |
| Trustworthiness | 25 | 0.64 | [0.55;0.72] | 0.17 | [-0.02;0.37] | 0.59 | [0.46;0.71] |
|  | 50 | 0.51 | [0.40;0.61] | 0.13 | [-0.05;0.33] | 0.42 | [0.27;0.57] |
|  | 100 | 0.44 | [0.32;0.56] | 0.43 | [0.26;0.59] | 0.28 | [0.09;0.47] |
|  | 200 | 0.55 | [0.45;0.64] | 0.47 | [0.33;0.61] | 0.41 | [0.27;0.56] |
|  | 400 | 0.72 | [0.66;0.78] | 0.71 | [0.61;0.79] | 0.72 | [0.62;0.80] |
|  | 800 | 0.78 | [0.73;0.83] | 0.76 | [0.67;0.83] | 0.73 | [0.63;0.81] |

| **Social Characteristic** | **Exposure** | **ICC** | **95% CIs** | **F** | **95% CIs** | **M** | **95% CIs** |
| --- | --- | --- | --- | --- | --- | --- | --- |
| Educatedness | 25 | 0.21 | [0.04;0.37] | 0.30 | [0.11;0.48] | 0.29 | [0.10;0.47] |
|  | 50 | 0.50 | [0.39;0.61] | 0.14 | [-0.04;0.34] | 0.00 | [-0.24;0.25] |
|  | 100 | 0.37 | [0.23;0.50] | 0.45 | [0.28;0.60] | 0.38 | [0.19;0.55] |
|  | 200 | 0.53 | [0.42;0.63] | 0.54 | [0.40;0.67] | 0.19 | [-0.04;0.41] |
|  | 400 | 0.68 | [0.60;0.75] | 0.75 | [0.67;0.83] | 0.59 | [0.46;0.71] |
|  | 800 | 0.73 | [0.67;0.79] | 0.81 | [0.74;0.87] | 0.58 | [0.44;0.70] |
| Poshness | 25 | 0.36 | [0.22;0.49] | 0.27 | [0.08;0.45] | 0.20 | [-0.01;0.40] |
|  | 50 | 0.48 | [0.37;0.59] | 0.31 | [0.14;0.48] | 0.32 | [0.13;0.50] |
|  | 100 | 0.57 | [0.47;0.67] | 0.47 | [0.31;0.62] | 0.42 | [0.25;0.59] |
|  | 200 | 0.69 | [0.62;0.76] | 0.73 | [0.63;0.81] | 0.65 | [0.53;0.75] |
|  | 400 | 0.79 | [0.73;0.83] | 0.76 | [0.68;0.84] | 0.75 | [0.67;0.83] |
|  | 800 | 0.74 | [0.68;0.80] | 0.74 | [0.65;0.82] | 0.75 | [0.67;0.83] |
| Professionalism | 25 | 0.00 | [-0.16;0.17] | 0.00 | [-0.14;0.17] | 0.11 | [-0.07;0.31] |
|  | 50 | 0.37 | [0.22;0.50] | 0.45 | [0.28;0.61] | 0.15 | [-0.09;0.38] |
|  | 100 | 0.38 | [0.25;0.50] | 0.43 | [0.28;0.58] | 0.37 | [0.21;0.53] |
|  | 200 | 0.68 | [0.61;0.75] | 0.77 | [0.69;0.84] | 0.57 | [0.44;0.69] |
|  | 400 | 0.73 | [0.66;0.79] | 0.77 | [0.69;0.84] | 0.69 | [0.59;0.78] |
|  | 800 | 0.85 | [0.81;0.88] | 0.88 | [0.84;0.92] | 0.78 | [0.70;0.85] |

*Supplementary Table 3: Tables showing the correlation coefficients associated with Figure 2 in the main manuscript. Correlation coefficients and 95% CIs are provided across all voices and for male and female voices respectively.*

| **Physical Characteristic** | **Exposure** | **Pearson's *r*** | **95% CIs** | **F** | **95% CIs** | **M** | **95% CIs** |
| --- | --- | --- | --- | --- | --- | --- | --- |
| Gender | 25ms | 0.98 | [0.97;0.99] | 0.80 | [0.67;0.88] | 0.69 | [0.51;0.81] |
|  | 50ms | 0.99 | [0.98;0.99] | 0.82 | [0.70;0.89] | 0.87 | [0.79;0.93] |
|  | 100ms | 0.99 | [0.99;0.99] | 0.88 | [0.79;0.93] | 0.89 | [0.82;0.94] |
|  | 200ms | 0.99 | [0.99;1.00] | 0.91 | [0.85;0.95] | 0.92 | [0.86;0.95] |
|  | 400ms | 1.00 | [0.99;1.00] | 0.94 | [0.89;0.96] | 0.95 | [0.91;0.97] |
| Age | 25ms | 0.75 | [0.65;0.83] | 0.64 | [0.43;0.78] | 0.65 | [0.45;0.78] |
|  | 50ms | 0.80 | [0.71;0.86] | 0.49 | [0.25;0.68] | 0.83 | [0.71;0.90] |
|  | 100ms | 0.85 | [0.78;0.89] | 0.65 | [0.45;0.79] | 0.84 | [0.73;0.90] |
|  | 200ms | 0.86 | [0.80;0.91] | 0.70 | [0.52;0.82] | 0.87 | [0.79;0.93] |
|  | 400ms | 0.91 | [0.87;0.94] | 0.85 | [0.75;0.91] | 0.86 | [0.76;0.92] |
| Health | 25ms | 0.57 | [0.42;0.69] | 0.40 | [0.14;0.61] | 0.45 | [0.20;0.65] |
|  | 50ms | 0.73 | [0.62;0.81] | 0.62 | [0.42;0.77] | 0.76 | [0.61;0.86] |
|  | 100ms | 0.71 | [0.60;0.80] | 0.52 | [0.29;0.70] | 0.71 | [0.53;0.82] |
|  | 200ms | 0.80 | [0.71;0.86] | 0.76 | [0.61;0.86] | 0.81 | [0.69;0.89] |
|  | 400ms | 0.92 | [0.88;0.94] | 0.92 | [0.87;0.96] | 0.90 | [0.82;0.94] |

| **Trait Characteristic** | **Exposure** | **Pearson's *r*** | **95% CIs** | **F** | **95% CIs** | **M** | **95% CIs** |
| --- | --- | --- | --- | --- | --- | --- | --- |
| Attractiveness | 25ms | 0.71 | [0.60;0.80] | 0.56 | [0.34;0.73] | 0.33 | [0.05;0.55] |
|  | 50ms | 0.24 | [0.05;0.42] | 0.50 | [0.26;0.68] | 0.36 | [0.09;0.58] |
|  | 100ms | 0.65 | [0.52;0.75] | 0.49 | [0.24;0.67] | 0.66 | [0.46;0.79] |
|  | 200ms | 0.68 | [0.56;0.77] | 0.80 | [0.67;0.88] | 0.55 | [0.32;0.72] |
|  | 400ms | 0.89 | [0.84;0.92] | 0.90 | [0.84;0.94] | 0.84 | [0.74;0.91] |
| Dominance | 25ms | 0.42 | [0.25;0.57] | 0.00 | [-0.28;0.28] | 0.26 | [-0.02;0.50] |
|  | 50ms | 0.49 | [0.32;0.62] | 0.12 | [-0.16;0.39] | 0.43 | [0.17;0.63] |
|  | 100ms | 0.58 | [0.43;0.69] | 0.54 | [0.31;0.71] | 0.39 | [0.12;0.60] |
|  | 200ms | 0.68 | [0.56;0.77] | 0.70 | [0.52;0.82] | 0.54 | [0.31;0.71] |
|  | 400ms | 0.79 | [0.70;0.85] | 0.79 | [0.66;0.88] | 0.78 | [0.64;0.87] |
| Trustworthiness | 25ms | 0.59 | [0.44;0.70] | 0.42 | [0.16;0.63] | 0.51 | [0.27;0.69] |
|  | 50ms | 0.63 | [0.49;0.73] | 0.56 | [0.33;0.72] | 0.51 | [0.27;0.69] |
|  | 100ms | 0.51 | [0.35;0.65] | 0.39 | [0.13;0.60] | 0.39 | [0.13;0.60] |
|  | 200ms | 0.71 | [0.59;0.79] | 0.62 | [0.41;0.77] | 0.64 | [0.44;0.78] |
|  | 400ms | 0.80 | [0.71;0.86] | 0.78 | [0.64;0.87] | 0.79 | [0.65;0.87] |

| **Social Characteristic** | **Exposure** | **Pearson's *r*** | **95% CIs** | **F** | **95% CIs** | **M** | **95% CIs** |
| --- | --- | --- | --- | --- | --- | --- | --- |
| Poshness | 25ms | 0.32 | [0.13;0.48] | 0.50 | [0.26;0.69] | 0.40 | [0.14;0.61] |
|  | 50ms | 0.39 | [0.21;0.54] | 0.55 | [0.32;0.72] | 0.59 | [0.38;0.75] |
|  | 100ms | 0.40 | [0.22;0.55] | 0.54 | [0.31;0.71] | 0.57 | [0.34;0.73] |
|  | 200ms | 0.65 | [0.51;0.75] | 0.72 | [0.55;0.83] | 0.67 | [0.48;0.80] |
|  | 400ms | 0.67 | [0.55;0.77] | 0.80 | [0.67;0.88] | 0.78 | [0.64;0.87] |
| Professionalism | 25ms | 0.37 | [0.18;0.53] | 0.32 | [0.05;0.55] | 0.42 | [0.16;0.63] |
|  | 50ms | 0.29 | [0.10;0.46] | 0.38 | [0.12;0.60] | 0.43 | [0.18;0.63] |
|  | 100ms | 0.58 | [0.44;0.70] | 0.58 | [0.36;0.74] | 0.70 | [0.52;0.82] |
|  | 200ms | 0.73 | [0.63;0.81] | 0.78 | [0.65;0.87] | 0.69 | [0.51;0.81] |
|  | 400ms | 0.84 | [0.77;0.89] | 0.91 | [0.84;0.95] | 0.88 | [0.80;0.93] |
| Educatedness | 25ms | 0.31 | [0.12;0.47] | 0.24 | [-0.04;0.49] | 0.41 | [0.14;0.62] |
|  | 50ms | 0.17 | [-0.02;0.36] | 0.07 | [-0.21;0.34] | 0.39 | [0.12;0.60] |
|  | 100ms | 0.32 | [0.13;0.48] | 0.38 | [0.12;0.60] | 0.24 | [-0.04;0.48] |
|  | 200ms | 0.46 | [0.29;0.60] | 0.64 | [0.44;0.78] | 0.48 | [0.23;0.67] |
|  | 400ms | 0.77 | [0.67;0.84] | 0.85 | [0.74;0.91] | 0.67 | [0.48;0.80] |

**Supplementary Analysis 2**

The different person characteristics are unlikely to be entirely orthogonal to one another. Below, Supplementary Figure 2 shows a matrix with exhaustive correlations between all exposure duration and all characteristics, which is reproduced in the supplementary materials for completeness. Broadly speaking, many characteristics are correlated with one another: There are strong correlations between all physical characteristics. Similarly, relatively strong correlations are apparent between trait characteristics and moderate-to-strong correlations are apparent between the social characteristics. Across characteristic “groups”, there are similarly moderate-to-strong correlations between physical and trait characteristics, as well as poshness. Correlations overall appear to be least strong and least consistent for between two social characteristics, educatedness and professionalism, and gender, age, and dominance.

There are also some hints about effects of exposure time (aside from ‘outlier’ data, e.g., impressions of attractiveness after 50ms of exposure): With, for example, correlations for between attractiveness, trustworthiness, and the three social characteristics increasing over time. These effects should be treated with caution: Correlations are based on mean ratings per item and interrater agreement for these particular characteristics was low for the shorter exposure durations. Thus, mean ratings per item for exposure durations with low agreement are likely to be very noisy at best.

*Supplementary Figure 1: Correlation matrix showing the correlation coefficients for all characteristics and exposure durations.*


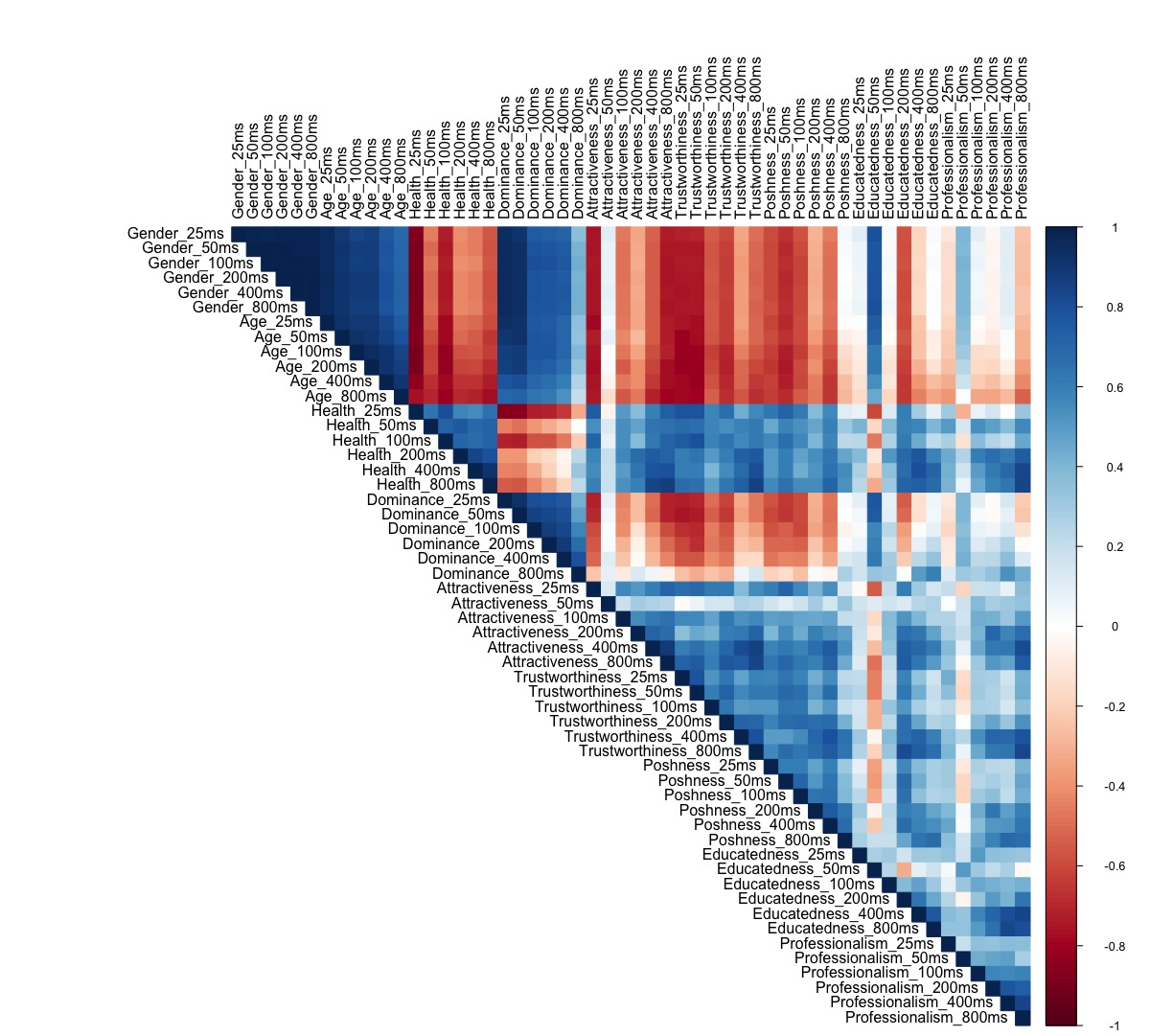


**Supplementary Analysis 3: Voice gender effects for interrater agreement**

ICCs by voice gender across all person characteristics and exposure times are plotted in Supplementary Figure 1. As discussed in the main text, ICCs are generally somewhat lower gender computes by gender. The figure below also shows that 95% CIs for male vs female voices tend to overall for all exposure durations for most person characteristics, such that there are no pronounced gender effects in the data. A notable exception to this pattern are the impressions of dominance, where agreement for male voices is substantially higher from earlier on, after 50 and 100ms of exposure, than for female voices. Otherwise, CIs do not overlap for ICCs for male and female voices for age impressions formed after 50ms of exposure and for trustworthiness impressions formed after 25ms of exposure.

*Supplementary Figure 2: Plot of time course for all 9 person characteristics by gender. Dashed lines show the ICC across time for male voices, continuous lines show the ICC across time for male voices. Shaded bands show the 95% confidence intervals.*


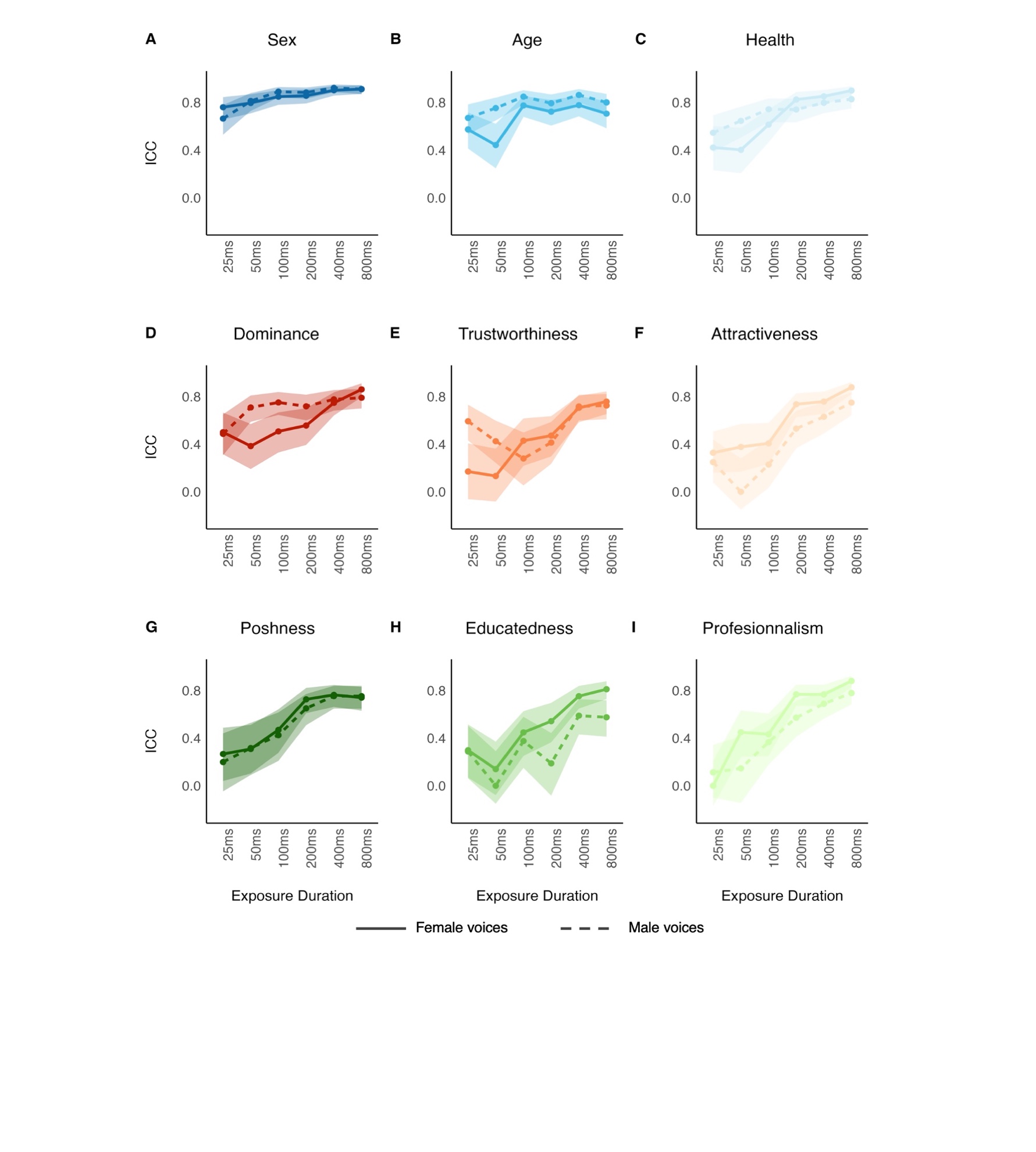

Supplement: sj-docx-1-pss-10.1177_09567976231161565 – Supplemental material for The Time Course of Person Perception From Voices: A Behavioral Study [file sj-docx-1-pss-10.1177_09567976231161565.docx]
